# Total Rehabilitation Using Adhesive Dental Restorations in Patients with Severe Tooth Wear: A 5-Year Retrospective Case Series Study

**DOI:** 10.3390/jcm12165222

**Published:** 2023-08-10

**Authors:** Álvaro Ferrando Cascales, Salvatore Sauro, Ronaldo Hirata, Daniela Astudillo-Rubio, Raúl Ferrando Cascales, Rubén Agustín-Panadero, Andrés Delgado-Gaete

**Affiliations:** 1 Department of Biomaterials Engineering, Faculty of Medicine, UCAM, Universidad Católica de Murcia, Campus Los Jerónimos, 135 Guadalupe, 30107 Murcia, Spain; aferrando@ucam.edu (Á.F.C.); rferrando@ucam.edu (R.F.C.); 2 Dental Biomaterials and Minimally Invasive Dentistry, Department of Dentistry, University CEU Cardenal Herrera, C/Santiago Ramón y Cajal, s/n, Alfara del Patriarca, 46115 Valencia, Spain; salvatore.sauro@uchceu.es; 3 Department of Therapeutic Dentistry, I. M. Sechenov First Moscow State Medical University, 119146 Moscow, Russia; 4 Department of Biomaterials and Biomimetics, New York University College of Dentistry, New York, NY 10010, USA; rh1694@nyu.edu; 5 Division of Prosthodontics, School of Dentistry, Universidad Católica de Cuenca, Cuenca 010107, Ecuador; dastudillor87@gmail.com (D.A.-R.); andydg86@gmail.com (A.D.-G.); 6 Prosthodontic and Occlusion Unit, Department of Stomatology, Faculty of Medicine and Dentistry, Universitat de València, 46010 Valencia, Spain

**Keywords:** increasing vertical dimension, minimally invasive dentistry, tooth wear, ceramic veneers, lithium disilicate, composite

## Abstract

Introduction: Currently, there is little clinical evidence to support the medium- and long-term survival and clinical performance of ultraconservative approaches using adhesive restorations in full-mouth restorations. The aim of this case series study was to evaluate the medium-term clinical performance of anterior and posterior adhesive restorations applied with direct and indirect techniques using resin composites and glass-ceramic-based materials. Materials and Methods: The inclusion criteria were an esthetic problem as the main reason for consultation and severe generalized wear of grade 2 to 4 according to the Tooth Wear Evaluation System (TWES 2.0). In addition, at each follow-up appointment, patients were required to submit a clinical-parameter-monitoring record according to the modified United States Public Health Service (USPHS) criteria. Results: Eight patients with severe tooth wear were treated through full rehabilitation in a private dental clinic in Spain by a single operator (AFC). A total of 212 restorations were performed, which were distributed as follows: 66 occlusal veneers, 26 palatal veneers and 120 vestibular veneers. No signs of marginal microleakage or postoperative sensitivity were observed in any occlusal, vestibular and/or palatal restoration after the follow-up period. The estimated survival rate of the 212 restorations was 90.1% over 60 months of observation, with a survival time of 57.6 months. Only 21 restorations had complications, which were mostly resolved with a direct composite resin. The dichotomous variables of the restoration type (posterior veneer, anterior veneer) and the type of restored tooth (anterior, posterior) were the risk predictors with statistically significant influences (*p* < 0.005) on the survival of the restorations. Conclusion: According to the results of this study, there is a significantly higher risk of restorative complications in posterior teeth compared to anterior teeth. Also, it can be concluded that the indication of adhesive anterior and posterior restorations is justified in the total oral rehabilitation of patients with severe multifactorial tooth wear, as they are associated with a low risk of failure.

## 1. Introduction

The current therapeutic approaches for the treatment of patients with severe tooth wear deriving from minimally interventionist dentistry base their clinical application on the excellent biomimetics and optimal mechanical and optical properties of both silica-based glass ceramics and composite resin-based restorations. Due to their physical characteristics, these materials achieve adequate adhesive strength when combined with different contemporary adhesive systems [1,2,3,4,5,6,7]. Due to the great development that adhesive dentistry has undergone, teeth affected by wear require minimal or no tooth preparation to achieve optimal aesthetic and functional results, with minimal sacrifice of the remaining structure by increasing the vertical dimension of occlusion (VDO) [8].

Dentition that has been subject to severe tooth wear because of chemical and/or mechanical causes requires interceptive dental procedures to limit future caries or endodontic treatments. These procedures are encompassed within full oral rehabilitations that frequently involve an increase in VDO [8,9,10].

Regarding material selection, lithium disilicate has a high long-term stability and better performance than composite resins [11,12]. However, in most studies, the financial means of patients are not considered. Although it is certainly necessary to assume a medium- to long-term re-intervention when choosing a composite resin, this is a feasible option from both a biological and economic point of view [13,14,15,16]. Several techniques using direct/indirect restorations of composite, ceramic or hybrid CAD/CAM materials to reconstruct worn teeth and restore a functional occlusal pattern in a stable mandibular position have been described [2,7,9,11,12].

In patients with severe dental wear, etiology is a risk factor for the restorative treatment if it is not controlled. For example, patients with occlusal parafunction may exceed the fatigue limits of different materials, which will increase the risk of fractures, or wear of chemical origin may also compromise marginal integrity and increase microleakage [1,9].

Currently, there is little clinical evidence to support the medium- and long-term survival and clinical performance of ultraconservative approaches using adhesive restorations in full-mouth restorations [15,16,17,18,19]. The aim of this study was to evaluate the medium-term clinical performance of anterior and posterior adhesive restorations performed with direct and indirect techniques using composite resins and glass-ceramic-based materials, both feldspathic and lithium disilicate, applied in patients with severe tooth wear who required an interdisciplinary approach with increased VDO.

## 2. Materials and Methods

The study protocol was approved by the Catholic University of Cuenca (Cuenca, Ecuador) Ethics Committee for Research Involving Humans (CEISH-UCACUE-2023-037). The study protocol complied with the guidelines established in the Declaration of Helsinki. All study participants received complete information on the objectives of the study, the procedures involved, alternative treatment options and the risks involved. All study participants gave informed consent to participate in the study.

The inclusion criteria were an esthetic problem as the main reason for consultation and severe generalized wear of grade 2 to 4 according to the Tooth Wear Evaluation System (TWES 2.0), which offers a complete taxonomy of tooth wear [20], including previous indirect restorations in the anterior and posterior sectors and patients who had received restorative treatment for at least 5 years. The patients’ medical records had to include initial and final photographs, type IV plaster models mounted on a semi-adjustable articulator (Panadent magentic PSH, Colton, CA, USA) or digital models with the initial and final VDO after treatment.

Finally, a detailed description of the teeth treated, as well as the materials used in each restoration, can be found in Refs. [7,8], including marginal adaptation, surface roughness, restoration fracture, tooth fracture, secondary caries and postoperative sensitivity. No exclusions were made based on the following factors: the etiology of wear (severe bruxism, gastroesophageal reflux, etc.) and orthodontic and/or endodontic treatment prior to the restorative process.

All patients were treated by an experienced operator, Álvaro Ferrando Cascales, DDS, PhD (AFC), using a semi-additive restorative approach that included the following clinical sequence:

1. An esthetic diagnosis using Digital Smile Design (DSD) [21].
2. The determination of VDO by means of an articulator set-up of the maxilla with a Kois dentofacial analyzer [22] (Panadent magentic PSH, Colton, CA, USA) and of the intermaxillary relations with the power centric manual induction technique [5].
3. Posterior sector: the diagnostic wax-up, fabrication and cementation of occlusal restorations in maxillary and/or mandibular posterior teeth to achieve a balanced occlusion with bilateral and simultaneous posterior contacts, thus restoring an adequate height to the lower facial third [2,3,4].
4. Anterior guide: treatment using maxillary palatal veneers without dental preparation and anteroinferior vestibular veneers for the provision of anterior guidance [3,4].
5. Final esthetics: anterosuperior vestibular veneers to achieve our final esthetic goals planned in the diagnostic process.

The criteria for exclusion were patients with localized tooth wear of grade 1 according to the TWES 2.0 criteria, periodontitis, severe gingival inflammation and a lack of cooperation regarding hygiene instructions.

Clinical and photographic information from the medical records was collected independently by two investigators, Andrés Delgado-Gaete, DDS, MsC, and Daniela Astudillo-Rubio, DDS, MsC (ADG, DAR), from January to October 2022. The clinical characteristics collected from the patients were sex, age, TWES 2.0 classification, whether or not they received orthodontic treatment, the type of treatment per tooth (occlusal veneer, facial veneer or palatal veneer), parafunction, the type of failure, time to failure and the solution (Table 1).

The survival rate was the main outcome variable, and the evaluation form for this variable was determined by the survival time. It was defined as the time elapsed from the successful adjustment of the restoration to the time when the restoration and/or restored tooth presented a failure that required dental intervention. For the evaluation, survival failures were considered as absolute when they met the following scores, according to the modified USPHS criteria [23,24]: a fracture restoration score of 2 (minor chipping on the restoration (1/4 of the restoration)), 3 (moderate chipping on the restoration (1/2 of the restoration)), 4 (severe chipping (3/4 of the restoration)) or 5 (debonding of the restoration); a secondary caries score of 1 (caries evidently continuous with the margin); a tooth fracture score of 2 (minor chipping on the tooth (1/4 of the crown)), 3 (moderate chipping of the tooth (1/2 of the crown)) or 4 (crown fracture near the cementum enamel line (extraction)); a surface roughness score of 2 (rough, cannot be refinished) or 3 (surface deeply pitted, irregular grooves); and a postoperative sensitivity score of 1 (slight sensitivity).

Survival analyses were performed with statistical software (SPSS 24.0; SPSS Inc., Chicago, IL, USA). Kaplan–Meier and log-rank (Mantel-Cox) tests were used to obtain the estimated survival and failure rates of the restorations at various time intervals, with the following variables: the restoration material (composite and ceramic), treatment type (posterior veneer and anterior veneer), parafunction (yes, no), TWES index (2, 3, 4), occlusion type (classes I, II, III), dental arch (upper, lower) and orthodontic treatment (yes, no). The univariate models were adjusted for each potential predictor using a Cox regression model, transforming the variables into dichotomous variables and including the variable patient as a random effect.

## 3. Results

This retrospective case series study included eight patients with a mean age of 46.6 years, or six men with a mean age of 45.5 years and two women with a mean age of 50 years.

**Figure 1:**
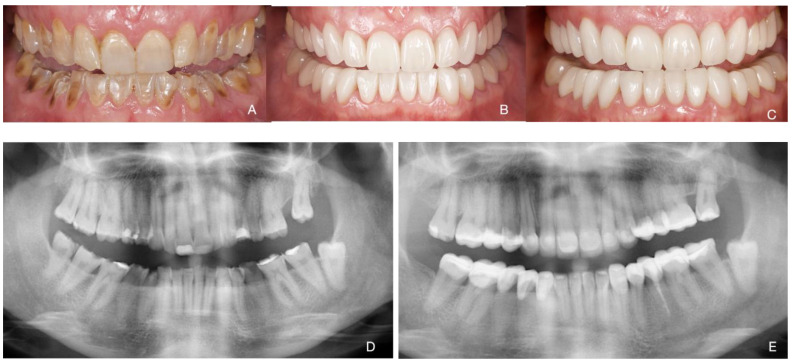
Patient 1. Generalized extreme pathological tooth wear (TWES 2.0). (**A**) Intraoral initial aspect of one Sjogren’s syndrome diagnosed. Deep-bite occlusal relationships and severely worn anterior teeth and severe tetracycline staining can be appreciated. (**B**) Intraoral aspect after orthodontic and restorative treatment, emphasizing great aesthetic and functional improvement. Occlusal-vestibular veneers from 1.6 to 2.6 and from 3.1 to 4.2. In the posteroinferior sectors (3.3–3.7 and 4.3–4.7), CAD-CAM nanoceramic resins were used. (**C**) A 5-year follow-up photograph. Note the maintenance of occlusal contact relationships, although there is slight wear of the posteroinferior sectors, as seen in the flattening of the cusps. (**D**) Initial orthopantomography. (**E**) Final orthopantomography showing the supplement of all teeth with an endodontic treatment in 4.5 due to previous necrosis.

**Figure 2:**
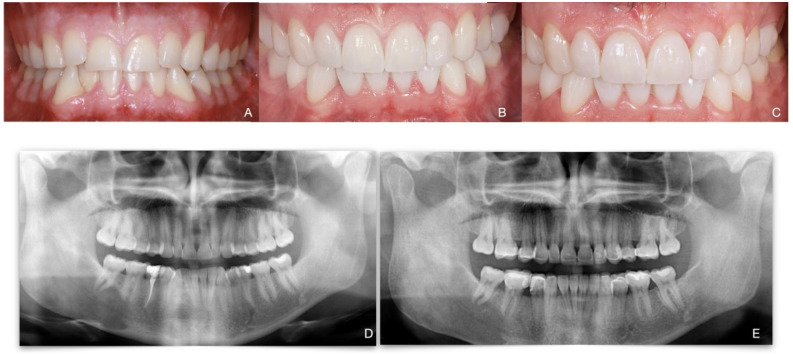
Patient 2. Generalized severe pathological tooth wear (TWES 2.0). (**A**) Pre-orthodontic intraoral situation showing a class II, division 2 occlusal relationship accompanied by severe crowding in the mandible. (**B**) Occlusion after orthodontic and restorative treatment, showing considerable aesthetic and functional improvement. (**C**) A 5-year follow-up photograph to underline the maintenance of occlusal contact relationships. Slight chronic gingivitis due to plaque accumulation can be observed. (**D**) Initial orthopantomography. (**E**) Final orthopantomography showing supplementation from 1.6 to 2.6 and from 3.6 to 4.6 by means of occlusal and vestibular lithium disilicate veneers.

**Figure 3:**
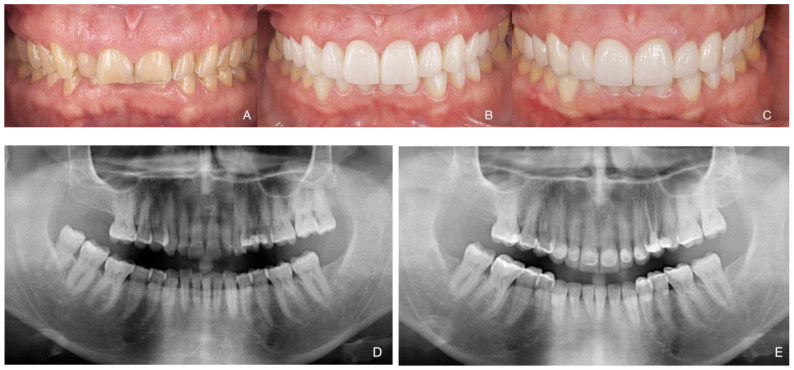
Patient 3. Generalized severe pathological tooth wear (TWES 2.0). (**A**) Intraoral situation: deep bite and occlusal relationships shown by a patient with severely worn anterior teeth. (**B**) Occlusion after orthodontic and restorative treatment, indicating aesthetic and functional improvement. Felspathic vestibular veneers from 1.5 to 2.5, CAD-CAM nanoceramic palatal veneers from 1.3 to 2.3, lithium disilicate veneers from 3.3 to 4.3 and a direct stamped composite resin in the posteroinferior sectors (3.4–3.6 and 4.4–4.6). (**C**) A 5-year follow-up photograph. Note the maintenance of occlusal contact relationships. (**D**) Initial orthopantomography. (**E**) Final orthopantomography showing the supplementation of all teeth with an endodontic treatment in 1.4 due to previous necrosis.

Four patients (50%) received orthodontic treatment (Figure 1, Figure 2 and Figure 3 prior to the restorative process with the aim of improving their sagittal, transverse and vertical maxillomandibular relations, as well as dental alignment. Six patients (75%) also presented with occlusal parafunction.

A total of 212 restorations were performed, which were distributed as follows: 66 occlusal veneers, 26 palatal veneers and 120 vestibular veneers.

**Figure 4:**
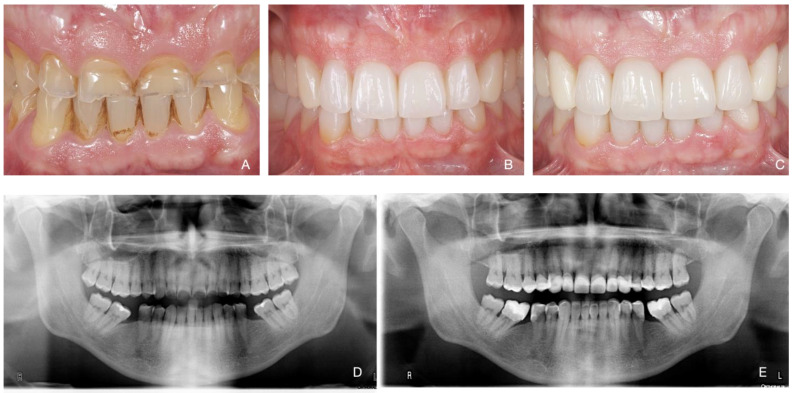
Patient 4: Generalized severe pathological tooth wear (TWES 2.0). (**A**) Intraoral situation: inverted occlusal plane shown for a patient with extremely worn anterior teeth associated with a compensatory extrusion of the mandibular anterior teeth. (**B**) Occlusion after treatment, highlighting aesthetic and functional improvement. (**C**) A 5-year follow-up photograph showing excellent periodontal health with the total absence of recession, highlighting the maintenance of occlusal contact relationships. (**D**) Initial orthopantomography. (**E**) Final orthopantomography showing the supplementation of all teeth without endodontic treatments. Felspathic vestibular veneers from 1.2 to 2.2, CAD-CAM nanoceramic resin palatal veneers from 1.3 to 2.3, and occlusal and vestibular veneers on 1.5, 1.4, 2.4 and 2.5. Finally, lithium disilicate veneers from 3.5 to 4.5 and 3.7, 4.7.

**Figure 5:**
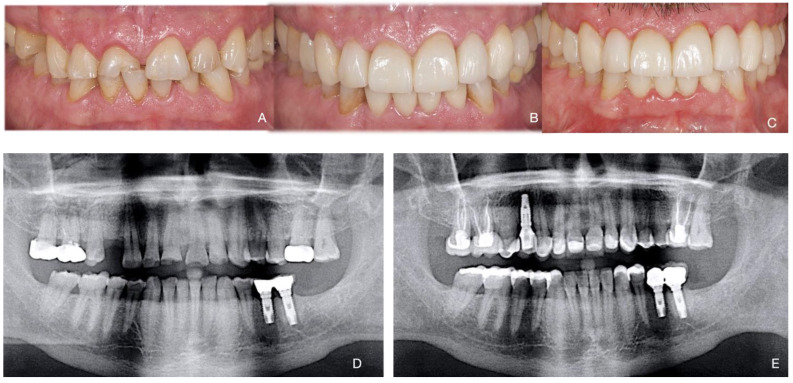
Patient 5: Generalized severe pathological tooth wear (TWES 2.0). (**A**) Intraoral situation: edge-to-edge bite and occlusal relationships shown by a patient with severely worn anterior teeth, (**B**) Intraoral appearance at 1 year. The aesthetic and functional improvement is remarkable, although there is considerable plaque accumulation. (**C**) Follow-up photograph at 5 years, showing acute gingivitis, which was treated successfully with a tartrectomy and the reinforcement of hygienic measures. (**D**) Initial orthopantomography. (**E**) Final orthopantomography showing the supplementation of all teeth. Lithium disilicate veneers from 1.4 to 2.3, CAD-CAM nanoceramic palatal veneers from 1.3 to 2.3, occlusal and vestibular direct composites on 1.5 and 2.5. Lithium disilicate veneers from 3.2 to 4.2, direct stamped composite resin in the posteroinferior sectors (3.3–3.4 and 4.3–4.7). One implant in 1.5 and the change of the prosthesis on implants 3.5 and 3.6 can be noted. Finally, we must mention a root canal treatment applied the teeth that had previous crowns.

**Figure 6:**
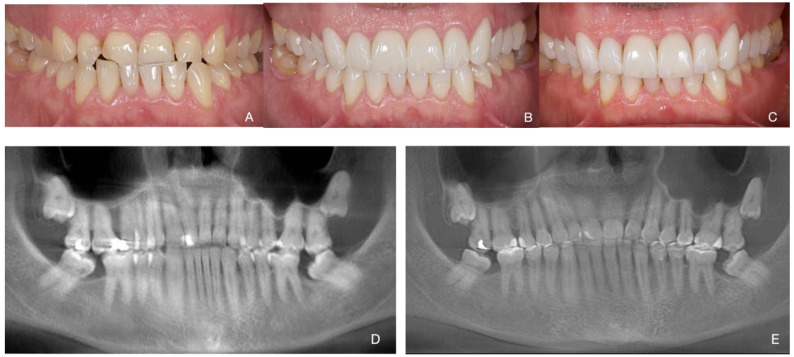
Patient 6: Generalized severe pathological tooth wear (TWES 2.0). (**A**) Intraoral situation: edge-to-edge bite and occlusal relationships shown by a patient with severely worn anterior teeth. A compensatory extrusion of the mandibular teeth can be appreciated (**B**) Occlusion after treatment, showing considerable esthetic and functional improvement. (**C**) Follow-up photograph at 5 years just after bicarbonate spray treatment, showing the maintenance of periodontal health at 5 years. (**D**) Initial orthopantomography. (**E**) Final orthopantomography showing facial veneers from 1.6 to 2.6 and free-hand direct composites in the mandible (3.6–4.6).

The occlusal and palatal veneers were fabricated with a CAD-CAM milled indirect composite resin (Cerasmart.GC, Tokyo, Japan). The vestibular veneers were fabricated with a classic feldspathic ceramic (Creation, Creation Willi Geller International GmbH, Meiningen, Germany) and reinforced with lithium disilicate (E.max Press, Ivoclar Vivadent, Schaan, Liechtenstein). The adhesive cementation of the restorations was performed with a light-cured resin cement (Calibra veneer bleach, Dentsply sirona. Konstanz, Germany) (Figure 4, Figure 5 and Figure 6).

The clinical parameters of the 212 restorations studied with the modified USPHS criteria for occlusal veneers, facial veneers and palatal veneers and their respective materials are described in Table 1.

No signs of marginal microleakage or postoperative sensitivity were observed in any of the occlusal, vestibular and/or palatal restorations after the follow-up period. The hypersensitivity presenting in some patients before treatment was efficaciously eliminated upon finishing the restorations. The surface roughness of all the restorations was within the USPHS score range 0 and 1, and no failures were considered in the survival analysis.

The fracture of the restoration, according to the USPHS criteria, represented the highest number of complications (18 of 212). Seventeen restorations showed slight chipping of 1/4 of the restoration (USPHS criteria, restoration score 2) which required clinician intervention, and the defects were repaired with a composite resin. One restoration had decementation (USPHS criteria, restoration score 5) and was recemented. We found one fractured tooth (USPHS criteria, restoration score 4), which required extraction and the placement of a dental implant to replace it. Finally, secondary caries lesions were found in two teeth (USPHS criteria, caries score 1), one of which required root canal treatment and composite resin repair, while the other required only composite resin repair.

**Scheme 1:**
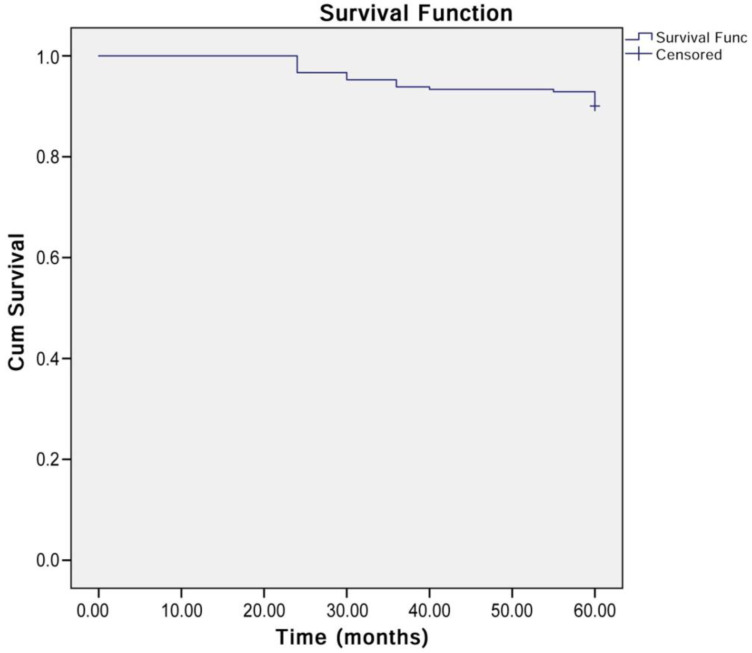
Kaplan–Meier plot showing the survival curves of all restorations at 60 months of follow-up.

The estimated survival rate of the 212 restorations was 90.1% during the 60 months of observation, with a survival time of 57.6 months. Only 21 restorations had complications requiring further intervention (Scheme 1). Most complications occurred at 2 and 5 years of follow-up, with estimated failure rates of 5% (10 restorations) and 6% (6 restorations), respectively, followed by a failure rate of 2% (4 restorations) in the 3rd year and a failure rate of 1% (1 restoration) in the 4th year of follow-up.

**Scheme 2:**
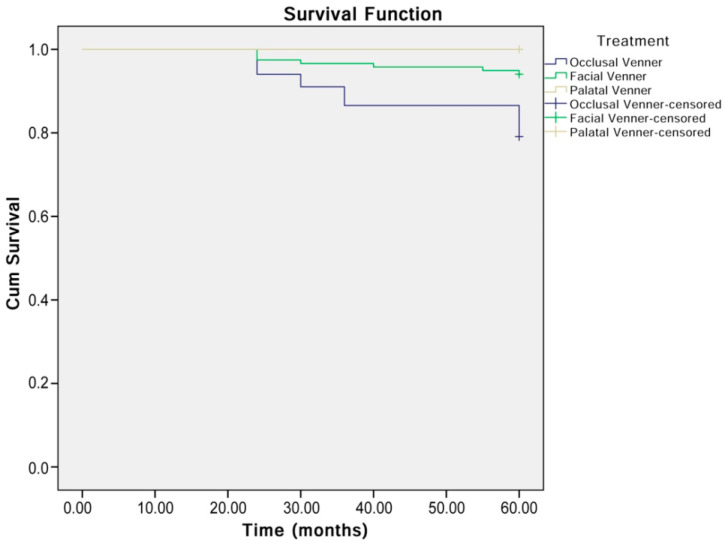
Kaplan–Meier plot showing the survival curves of different types of treatment.

**Scheme 3:**
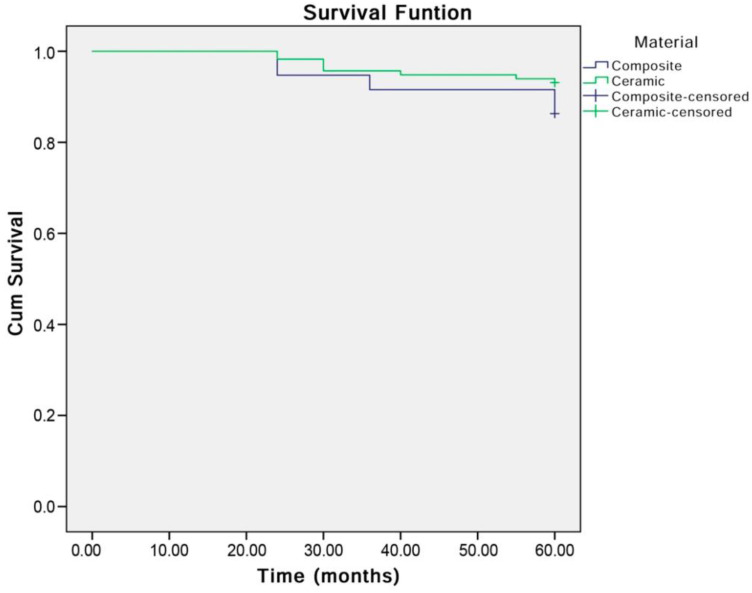
Kaplan–Meier plot showing the survival curves of different types of materials.

The type of treatment (occlusal veneer, facial veneer, palatal veneer) had a statistically significant influence on the estimated survival rate (*p* < 0.000). Occlusal veneers had an estimated survival rate of 79.1%, with rates of 94.1% for vestibular veneers and 100% for palatal veneers (Scheme 2). Composite resin restorations had a mean survival rate of 86.3%, while ceramic restorations had a survival rate of 93.1% (*p* = 0.107) (Scheme 3).

The variables studied, tooth wear classification, type of occlusion, dental arch, previous orthodontic treatment, and use of occlusal splints did not have a statistically significant influence on the estimated survival rate of the restorations at 60 months of follow-up (*p* > 0.05). Teeth with a generalized tooth wear index of grade 2 or 3 had estimated survival rates of 91.4% and 85.7%, respectively (*p* = 0.603). Restorations in patients with a class II occlusion had a survival rate of 81.8% versus 92.6% and 95.2% in patients with class I and III occlusions, respectively (*p* = 0.058). The survival rates for restorations placed in the upper and lower jaw were 89.7% and 90.5%, respectively (*p* = 0.828). Restorations placed in patients who received orthodontic treatment prior to the restorative phase had a survival rate of 88.7% versus 91.2% for patients who had not received orthodontic treatment (*p* = 0.540). Lastly, the survival of restorations in patients who wore occlusal bite guards was 91.8%, while in those who did not, it was 84.6%, (*p* = 0.138).

The univariate risk analysis of the predictors affecting the survival of restorations is shown in Table 1. The dichotomous variable of restoration type (posterior veneer, anterior veneer) was the risk predictor with a statistically significant influence (*p* < 0.005) on the survival of the restorations. The use of a bite guard, type of occlusion, dental arch and orthodontic treatment were not observed as predictor variables with statistically significant influences on the survival of the restorations.

## 4. Discussion

Consensus in clinical management guidelines and evidence-based recommendations for the choice of material and restoration type in this group of patients are limited [1,5,11,12,13,14,15,16]. Currently, the literature is sparse in terms of indications and scarce in evaluations of the survival rates and clinical performance of different types of materials and restorations used to perform full oral rehabilitations [7,9,10,11,12,13,14,15,16,17,18,19]. For example, the systematic review conducted by Mesko et al. in 2016 [18] did not render an open picture, concluding that there is no strong evidence to suggest that one material is better than another. Direct or indirect materials may be feasible options to restore severely worn teeth [2,14,15,16,17].

There is no doubt that in vivo clinical studies allow for the testing of intraoral conditions that cannot be fully reproduced in vitro, thus allowing for a true evaluation of the behavior of new materials and techniques currently being performed in patients with severe multifactorial tooth wear and, therefore, the extrapolation of specific indications. In this regard, it is worth noting the prospective trial conducted by Gúth JF et al. on 12 patients in 2020 [25], in which significantly less occlusal wear of lithium disilicate, as compared to CAD-CAM composites, was observed over a follow-up of only 2 years.

In our study, although retrospective, ceramic and composite materials (anterior and posterior) were equally evaluated over a controlled clinical follow-up of 5 years [6]. The high cumulative survival rates are similar or slightly lower than the results obtained in similar studies involving all types of adhesive restorations. Of note is the study by Torosyan A et al. published in 2022, with an overall survival rate of 99% at 6 years for 406 restorations [26]. The 6-year survival rates were 97.3% for direct composites (anterior–posterior); 98.2% for onlays, both composite and ceramic; and 100% for veneers, again both composite and ceramic (*p* > 0.05). No differences were found between the materials and locations of the restorations. The total of 19 technical complications included 14 partial fractures, 3 fissures, 1 wear, and 1 decementation. The USPHS evaluation showed good technical outcomes.

Nevertheless, our results, as in the case of Torosyan A et al., are in accordance with the main conclusion of the study conducted by Loomans. B and Opdam. N in 2018 [14]. Restorations, including composites and veneers or crowns, do not prevent wear processes; they simply modify the rate, location and nature of wear. Additionally, most restorations that are considered “definitive” may have a limited service life in cases with severe tooth wear due to bruxism and erosion. One of the most important aspects is the notion that possible treatment options and anticipated complications should be explained to patients in the informed consent process.

The literature is conclusive regarding the use of occlusal bite guards. Faus V et al., in 2020 [27], reported that bruxism patients who used bite guards showed a survival rate of 89.1% after 7 years, whereas the survival rate in bruxism patients who did not use guards was 63.9% (*p* < 0.05). In the study by Torosyan et al. [26], the presence or absence of bite guards did determine survival (*p* = 0.003). In our study, survival was 90.1% at 60 months of observation, and the application of an occlusal bite guard had a positive influence on the cumulative survival of restorations (hazard ratio 1.91; *p* < 0.149).

**Figure 7:**
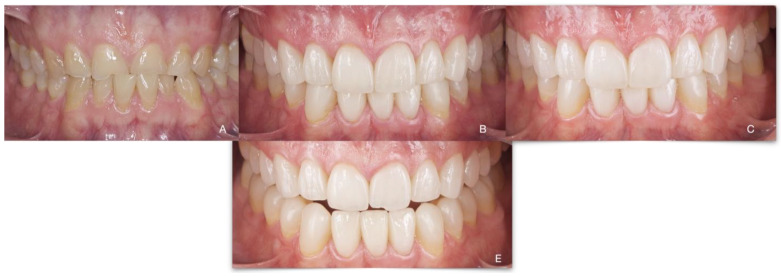

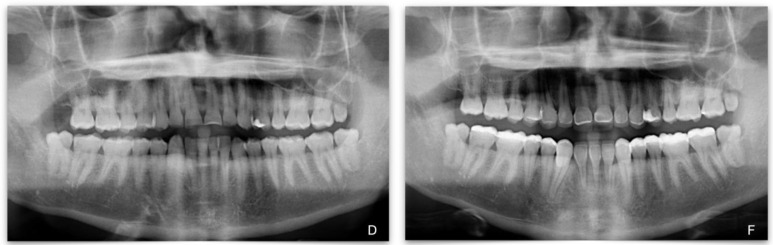
Patient 7. Generalized severe pathological tooth wear (TWES 2.0). (**A**) Intraoral situation: edge-to-edge bite and occlusal relationships shown by a patient with severely worn anterior teeth. We can observe a class III malocclusion that was solved with an extraction of the lower right central incisor (4.1) associated with a complete orthodontic treatment prior to the restorative phase. (**B**) Occlusion after orthodontic treatment (the extraction of 4.1 was necessary to alleviate crowding). Restorative treatment was performed with composites in the mandible and feldesphatics ceramics in the maxilla. The aesthetic and functional improvement is noteworthy. (**C**) Resolution of the fracture with a direct composite resin. Note the maintenance of occlusal contact relationships and periodontal health at 5 years. (**D**) Initial orthopantomography. (**E**) Follow-up at 4 years. Fracture of the incisal edge of the ceramic veneer on 2.1 due to trauma with a glass bottle. (**F**) Final orthopantomography showing direct stamped composite resin supplementation in the posteroinferior sectors (3.3–3.7 and 4.3–4.7) and feldesphatics veneers from 1.5 to 2.5 and on 4.2, 3.1 and 3.2.

**Figure 8:**
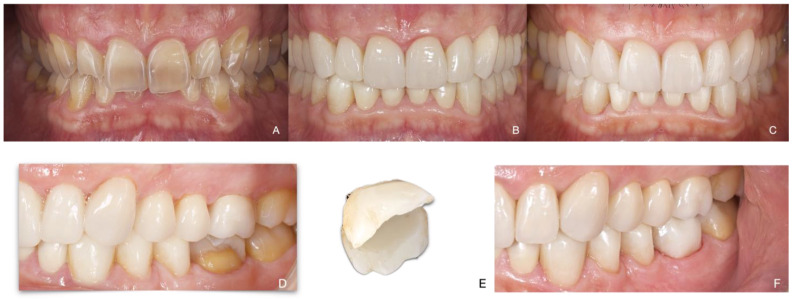
Patient 8. Generalized severe pathological tooth wear (TWES 2.0). (**A**) Intraoral situation: deep overbite and occlusal relationships shown by a patient with severely worn anterior teeth and severe tetracycline staining. (**B**) Occlusion after restorative treatment from 1.6 to 2.6 with feldsphatic veneers in the maxilla and from 3.2 to 4.2. CAD-CAM indirect nanoceramic resins were used for the palatal veneers (1.3–2.3) and in the mandible (occlusal–vestibular from 3.3 to 3.7 and from 4.3 to 4.7). From a patient perspective, the aesthetic and functional improvement is outstanding. (**C**) Follow-up photograph at 5 years. Note the maintenance of occlusal contact relationships and periodontal health, although there is recession in 1.3 and 1.2. (**D**) Fracture of CAD-CAM composite nanoceramic resin in 3.6 at 4 years. (**E**) Detail of the fractured fragment. (**F**) Re-bonding of the fractured fragment.

In our case series, all 212 restorations evaluated showed excellent clinical behavior at the 5-year follow-up, while only 18 restorations showed 1/4 chipping (USPHS criteria, restoration score 2). This very low failure rate is in accordance with the studies included in the systematic review by Mesko et al., 2016 [28], and the survival rate reported by Milosevic et al., 2016, at eight years [19]. The complications were easily repairable by the clinician (AFC) (Figure 7 and Figure 8), following an established evidence-based protocol [6,7,18,19]. This included 50-micron aluminum oxide sandblasting (Dento-prep. Ronvig, Daugaard, Denmark) for the composite resin and hydrofluoric acid etching (Porcelain etch. Ultradent Products, Inc., South Jordan, UT, USA) for the ceramic, followed by active cleaning with 37% orthophosphoric acid (Ultra-etch. Ultradent Products, Inc., South Jordan, UT, USA) for 2 min and the application of a silane-based bonding agent, which was heat-activated for one minute with an LED polymerization lamp (Smartlite Pro. Dentsply Sirona, Konstanz, Germany). Finally, a bonding agent belonging to a fourth-generation adhesive system (Heliobond, Ivoclar Vivaldent, Schaan, Liechtenstein) was applied.

The survival rate, based on the type of treatment performed (occlusal veneer, facial veneer, palatal veneer), also showed statistically significant differences (*p* < 0.05) with the estimated survival rate, being lower for occlusal veneers compared to vestibular and palatal veneers. This is probably because posterior restorations are subject to more fatigue and/or wear than anterior restorations [29].

The restoration of worn dentitions is widely described in the literature [1,2,3,4,5,6,7,8,9,10,11,12,13,14,15,16,17,18,19]. With the main advantage of adhesive restorations being the fact that a semi- or fully additive approach is adopted, the study by Fradeani et al., conducted in 2021, concluded that the cumulative survival rate recorded was 99.15%, with a ten-year survival probability of 96.5%. These remarkable results strongly support the use of a Minimally Invasive Preparation Procedure (MIPP) as a restorative option for severely worn dentitions [12].

In contrast, conventional classical tooth preparation approaches based on crown-type restorations compromise more of the already deteriorated tooth structure [30]. Although they have demonstrated equal long-term success, with 15–20-year survival rates ranging between 50 and 80% [31], they deviate from the guidelines established in the latest European consensus statement on the treatment of severely worn teeth [1] that advocate for the use of adhesive, direct or indirect techniques, which usually allow for a second chance in cases of the failure and/or wear of the previous restoration [32].

## 5. Conclusions

According to the results of this study, there is a significantly higher risk of restorative complications in posterior teeth compared to anterior teeth (*p* < 0.005). However, the best treatment for defective and/or partially fractured restorations is conservative management based on direct repair. Replacements should be limited to very extensive fractures that themselves compromise the restorations’ survival [33].

Considering the limitations of this study, including the sample size and great biological variability in wear, it can be concluded that the indication of anterior and posterior adhesive restorations is justified in the total oral rehabilitation of patients with severe multifactorial tooth wear.

The adhesive restorative approach, regardless of the material chosen, presents a low risk of failure at 5 years. The complications are sustainable in daily clinical practice.

## Figures and Tables

**Table 1:** Univariable comparisons of predictor factors for the failure of restorations.

Variable	No Failure	Failure	Hazard Ratio (95% CI), and *p*-Value (Cox Regression)
Posterior Veneer (indicator)	53	14	0.143 (CI 0.034–0.601) *p* = 0.008
Anterior Veneer (pacial, palatal)	138	6	
Composite, Direct/Indirect (indicator)	83	13	1.703 (CI 0.491–5.903) *p* = 0.401
Ceramic	108	7	
Bite Guard NO (indicator)	45	7	0.658 (CI 0.222–1.949) *p* = 0.450
Bite Guard YES	146	13	
TWES 2.0 2 (Indicator)	44	6	1.362 (CI 0.529–3.512) *p* = 0.522
TWES 2.0 3–4	147	14	
Class I	124	10	2.355 (CI 0.992–5.591) *p* = 0.052
Class II–III (indicator)	67	10	
Maxilla (indicator)	106	11	0.494 (CI 0.177–1.380) *p* = 0.179
Mandible	85	9	
Orthodontic Treatment YES (indicator)	87	10	1.303 (CI 0.553–3.067) *p* = 0.545
Orthodontic Treatment NO	104	10	

## Data Availability

Information is available on request in accordance with any relevant restrictions (e.g., privacy or ethical).
